# Low serum 25-hydroxy vitamin D levels are associated with aggressive breast cancer variants and poor prognostic factors in patients with breast carcinoma

**DOI:** 10.20945/2359-3997000000062

**Published:** 2018-08-01

**Authors:** Arunkumar Karthikayan, Sathasivam Sureshkumar, Dharanipragada Kadambari, Chellappa Vijayakumar

**Affiliations:** 1 Jawaharlal Institute of Postgraduate Medical Education and Research Department of Surgery Pondicherry India Department of Surgery, Jawaharlal Institute of Postgraduate Medical Education and Research (JIPMER), Pondicherry, India

**Keywords:** 25-hydroxy vitamin D, carcinoma breast, prognostic factors, vitamin D metabolism, cancer prophylaxis

## Abstract

**Objective:**

This study was conducted to assess the serum 25-hydroxy (OH) vitamin D levels in patients with breast cancer compared to healthy controls and to identify its association with aggressive breast cancer phenotypes.

**Materials and methods:**

Serum 25-OH vitamin D levels of 78 breast cancer patients and 78 matched healthy controls were estimated using ELISA. The cases and controls were matched with respect to age, menopausal status, parity, weight, height and co-morbidities. Prognostic factors like grade of tumour, hormone receptor status, *HER2 neu* status and lymphovascular invasion were compared with 25-OH vitamin D levels.

**Results:**

The mean serum 25-OH vitamin D levels of cases were significantly lower compared to the controls (22.33 ± 8.19 vs. 37.41 ± 12.9 ng/mL; p = 0.0001). Patients with higher grades of tumour, non-luminal types of breast cancer and breast cancers with estrogen receptor negativity had significantly lower serum 25-OH vitamin D levels than their opposing groups. Patients with excellent and good Nottingham's prognostic Index (NPI) had significantly higher serum 25-OH vitamin D levels than the moderate and poor NPI groups.

**Conclusion:**

Newly diagnosed breast cancer patients have significantly lower serum 25-OH vitamin D levels than healthy controls. Lower level of serum 25-OH vitamin D correlates with aggressive breast cancer phenotypes.

## INTRODUCTION

Breast cancer is the commonest malignancy among women worldwide (1). Vitamin D has a wide range of immunogenic and anti-proliferative action throughout the body, in addition to its well-known endocrine actions. Vitamin D deficiency has been correlated with increased incidence of malignancies of breast, prostate, and colon (2). Suboptimal vitamin D levels are hypothesized to lead to unhindered cellular proliferation, angiogenesis, and metastasis (3). Suboptimal vitamin D levels in women diagnosed with breast cancer have been shown to correlate with poor overall survival and decreased distant disease free survival (4).

Though Western literature shows a correlative relationship between vitamin D status and increased incidence of breast cancer, its impact in the Indian population is of questionable value because India is a tropical country with abundant sunshine throughout the year. This study might form part of the missing links in the vitamin D and breast cancer scenario in India. The Indian and white European populations vary significantly in their characteristics, and a positive correlation in this study as in its Western counterparts might pave the way for larger observational studies defining the level of vitamin D deficiency in Asian Indians (5). If large-scale studies show a significant correlation, it might pave the way for better understanding in the management of breast cancer.

## MATERIALS AND METHODS

### Study design

This study was a hospital-based case control study conducted over a period of 2 years. All the newly diagnosed breast cancer patients presenting to a tertiary care center in South India were included in the study. The study was evaluated by the institute's ethics committee and a formal clearance was obtained (JIP/IEC/2012/26/324). The nature, methodology, and risks involved in the study were explained to the patients and informed consent was obtained. All the information collected was kept confidential and patients were given full freedom to withdraw at any point during the study. All provisions of the Declaration of Helsinki were followed in this study.

### Cases and controls

Newly diagnosed breast cancer patients not on any vitamin D supplementation for the past 6 months were included in the study. Patients who were diagnosed elsewhere or partially treated at outside hospitals and who had taken vitamin D supplementation in the previous 6 months were excluded from the study. Details regarding demographic parameters, parity, menopausal status, co-morbidities, family history of breast cancer, clinical features, height, weight, examination findings pertaining to breast carcinoma, and baseline laboratory parameters were collected.

The controls were selected from apparently healthy women volunteers accompanying the patients attending this institute after due informed written consent. The cases and controls were matched with respect to age, menopausal status, parity, weight, height, co-morbidities and renal function test, so that the confounding factors affecting the serum 25-OH vitamin D levels are avoided.

All the participants included were the residents of Tamil Nadu and Pondicherry states in South India with latitude of 11.1271° N- 11.9139° N, 78.6569° E- 79.8145° E. The climate in this part of country is classified as tropical wet and dry (megathermal) by the Köppen-Geiger system where the population gets sun exposure in most parts of the year. Additionally, the clothing habit of women in this part of the country enables them to get adequate sun exposure.

### Serum 25-OH vitamin D level estimation

The serum 25-OH vitamin D levels were estimated with DIA Source immunoassay KAP 1971 25-OH vitamin D Total ELISA Kits. The assay was calibrated to the ID-LC/MS-MS reference method. 25-OH vitamin D level &gel 30 ng/mL was considered sufficiency. Ten to < 30 ng/mL was taken as 25-OH vitamin D insufficiency and 0 to < 10 ng/mL as deficiency. The above cut-off values were selected based on the recommendation of Endocrine Society practice guidelines (6).

### Outcome parameters studied

The bold sample for serum 25-OH vitamin D level estimation was taken at the time of diagnosis confirmation by core needle biopsy. The blood sample was collected from the healthy female volunteers after matching at the outpatient department. The demographic characteristics were recorded including age, BMI, height, weight, and Hb. Status of menopause was recorded in both the groups. Details of all the breast cancer patients were discussed in the tumor board for making informed decisions regarding the treatment plans. The breast cancer patients’ biopsy reports were followed up after surgery. Details like grade of the tumor, hormone receptor status, *HER2 neu* status, lymphovascular invasion, and Nottingham's prognostic index (NPI) were collected and studied to compare the level of vitamin D and its association with the above parameters.

### Statistical analyses

The distribution of data on 25-OH vitamin D levels will be assessed by using the Kolmogorov-Smirnov test and accordingly expressed as the mean with standard deviation. Data were analyzed using SPSS software version 17. The 25-OH vitamin D levels between the two groups were compared by using chi-square test, and the mean 25-OH vitamin D level was compared using Student's t-test. Association between serum 25-OH vitamin D levels and various breast cancer prognostic parameters such as tumor stage, grade, histological type receptor status, etc., was analyzed using ANOVA and Student's t-test. A *p* value of < 0.05 was considered as statistically significant.

## RESULTS

All the newly diagnosed patients attending the breast cancer clinic were screened for inclusion in the study. Seventy-eight cases over a period of 2 years were included after satisfying the inclusion/exclusion criteria. Seventy-eight controls were studied after necessary matching as mentioned in the cases and control definition.

Demographic parameters like age, height, and weight in both control and breast cancer groups were comparable. The BMI was the same in both groups and fell within the normal range for all of the subjects and controls (Table 1).

**Table 1:** Comparison of baseline characteristics between cases and controls

Parameters	Controls (n = 78) (Mean ± SD)	Cases (n = 78) (Mean ± SD)	*p* value (Student *t* test)
Age (years)	45.8 ± 10.1	47.1 ± 10.7	0.439
BMI (kg/m^2^)	22 ± 2.3	22 ± 2.2	0.940
Height (cm)	162.6 ± 2.6	161.9 ± 2.7	0.133
Weight (kg)	58.2 ± 5.8	57.8 ± 5.4	0.660
Hemoglobin (g/dL)	12 ± 0.8	11.8 ± 0.8	0.184

## Base line vitamin D levels

Around 4% of the women in the breast cancer group were vitamin D deficient, whereas in the control group no women had vitamin D deficiency. Of the breast cancer patients, 82% had vitamin D insufficiency, in contrast to 35% in the control group (*p* < 0.001). Only 14% of patients had sufficient vitamin D levels compared to 65% of controls who had sufficient vitamin D levels (Table 2).

**Table 2:** Comparison of serum 25-OH vitamin D levels in cases and controls

25-OH vitamin D levels	Controls		Cases		*p* valuex
	N (%)	25(OH) vitamin D Level (ng mL) Mean ± SD	N (%)	25(OH) Vitamin D Level (ng/mL) Mean ± SD	
Deficiency	0	NA	3 (3.9%)	8.5 ± 1.5	NA
Insufficiency	27 (34.6%)	26 ± 2.8	64 (82%)	20.3 ± 4.7	< 0.001*
Sufficiency	51 (65.4%)	43.5 ± 10.8	11 (14.1)	38.1 ± 4.8	0.112*
Mean serum 25(OH) vitamin D level	78	37.4 ± 12.2	78	22.3 ± 8.2	0.0001**

* Chi-square test,

** Student *t* test.

## Vitamin D levels versus breast cancer prognostic parameters

An equal number of premenopausal and postmenopausal women were in the two groups. No significant difference was observed between serum 25-OH vitamin D levels in postmenopausal women and premenopausal women (20.13 ± 8.51 ng/mL vs. 23.7 ± 7.77 ng/mL; *p* = 0.06). Women who did not fall into the clear-cut category of premenopausal or postmenopausal were classified into the perimenopausal group, and seven of such women were not included in the analysis. No patients presented in stages I and IV. The clinical stage of a tumor did not bear any statistically significant relationship with the serum 25-OH vitamin D levels in this study. A statistically significant association (*p* = 0.001) was observed between serum 25-OH vitamin D levels and histological grades of breast cancer. Patients with poorly differentiated (grade 3) breast cancer had lower 25-OH vitamin D levels than patients with moderately (grade 2) and well-differentiated (grade 1) tumors (Table 3).

**Table 3:** Association between serum 25-OH vitamin D levels and breast cancer prognostic parameters

Breast cancer prognostic parameters		Serum 25(OH) vitamin D level (ng/ mL)	*p* value
Menopausal status	Premenopausal (n = 48)	23.7 ± 7.8	0.060*
	Postmenopausal (n = 23)	20.1 ± 8.5	
Stage of tumour	Stage I (n = 0)	0	0.598**
	Stage II A (n = 9)	24.8 ± 7.7	
	Stage IIB (n = 40)	21.3 ± 8	
	Stage IIIA (n = 25)	23.4 ± 9.2	
	Stage III B (n = 4)	21.1 ± 4.1	
	Grade 1 (n = 16)	29.5 ± 10.4	
Grade of tumour	Grade 2 (n = 53)	21.7 ± 6	0.001**
	Grade 3 (n = 9)	13.4 ± 3.7	

* Student's *t* test,

** ANOVA.

## Vitamin D levels versus NPI

NPI was used as a surrogate indicator for aggressiveness of a tumor. On analyzing the association with the individual prognostic category, 25-OH vitamin D levels did not show a significant association with the NPI. However, when the NPI was grouped to include various prognostic categories into two groups - Group I (index < 3.4) consisting of the excellent (index ≤ 2.4) and good prognostic categories (index ≤ 2.4-3.4) and Group II (index > 3.4) consisting of the moderate I (index 3.41-4.40), moderate II (index 4.41-5.4), and poor (index ≥ 5.41) prognostic categories - serum 25-OH vitamin D in Group II showed significantly lower mean concentration than in Group I (25.48 ± 8.86 ng/mL vs. 20.46 ± 7.24 ng/ml; *p* = 0.008) (Table 4).

**Table 4:** Association between serum 25-OH vitamin D levels and Nottingham's prognostic index (NPI)

Prognostic category	NPI Score	Serum 25 (OH) Vitamin D levels (ng/mL) Mean ± SD	*p* value
Excellent (n = 7)	≤ 2.4	28.6 ± 10.3	
Good (n = 22)	2.4 – 3.4	24.5 ± 8.4	
Moderate I (n = 21)	3.4 – 4.4	20.6 ± 7.5	0.075*
Moderate ll (n = 19)	4.4 – 5.4	19.9 ± 8.4	
Poor (n = 9)	> 5.4	21.3 ± 3.6	
Group I (n = 29)	≤ 3.4	25.5 ± 8.9	0.008**
(Excellent, good)			
Group II (n = 49)	> 3.4	20.5 ± 7.2	
(Moderate I & II, Poor)			

* ANOVA,

** Student's *t* Test.

## Vitamin D levels versus IHC molecular phenotypes

The molecular phenotypes of breast cancer were segregated using IHC status as surrogate markers. On analyzing the individual molecular phenotype, the vitamin D levels did not yield any significant association with any of the luminal A, luminal B, *HER2,* and basal phenotypes of the tumors. However, when the phenotypes were categorized into a non-luminal group *(HER2* and basal) and a luminal group (luminal A and luminal B), the non-luminal group had a significantly lower mean serum 25-OH vitamin D level as opposed to the luminal group (20.6 ± 8.1 vs. 24.3 ± 8 ng/mL; *p* = 0.045) (Table 5).

**Table 5:** Association between serum 25-OH vitamin D levels and molecular phenotypes of breast cancer

Molecular Phenotypes	Serum 25 (OH) Vitamin D Levels (ng/mL) Mean ± SD	p value
Luminal (n = 37)	24.3 ± 8	0.045*
Non-luminal (n = 41)	20.6 ± 8.1	
Luminal A (n = 25)	23.6 ± 7.4	
Luminal B (n = 12)	25.7 ± 9.4	0.206**
Her 2 (n = 15)	21 ± 9.1	
Basal (n = 26)	20.3 ± 7.6	

* Student's *t*Test,

** ANOVA.

## Vitamin D levels versus lymphovascular invasion and estrogen receptors

The group of patients with lymphovascular invasion in breast cancer had no significant association with lower mean vitamin D value compared to the group without lymphovascular invasion (*p* = 0.360). Patients with estrogen receptor positivity had significantly higher serum 25-OH vitamin D levels compared to the patients with negative estrogen receptors (24.28 ± 7.99 vs. 20.57 ± 8.08; *p* = 0.045) (Table 6).

**Table 6:** Association between serum 25-OH vitamin D Levels with histopathology parameters

Histopathology parameters		Serum 25 (OH) Vitamin D levels (ng/mL)	p value (Student *t* test)
Lymphovascular Invasion	Present (n = 17)	20.7 ± 7.2	0.360
	Absent (n = 61)	22.8 ± 8.5	
Estrogen Receptor	Present (n = 37)	24.3 ± 8	**0.045**
	Absent (n = 41)	20.6 ± 8.1	

## DISCUSSION

Multiple studies have shown associations between adequate vitamin D, circulating vitamin D levels, and a decreased incidence of malignancies of colon, breast, ovary, prostate, kidney, pancreas, etc. (3,4). It has been shown that a mean vitamin D level of 40 to 60 ng/mL will be able to prevent about three-fourths of the deaths from breast and colon cancer in the United States and Canada (2). Multiple laboratory tests on tissue cultures have shown that the malignant cell growth is inhibited by vitamin D metabolites, and there has been redifferentiation in some instances (3,4,7). The proposed mechanisms for the said action includes up-regulation of adherence and signaling between epithelial cells, contact inhibition of proliferation, differentiation, cell cycle stabilization, promotion of apoptosis, antineoangiogenesis, and down-regulation of glycogen synthase kinase 3 (GSK-3) that decreases the in-vitro proliferation of colorectal, pancreatic, prostate, and other cancer cells (7).

According to Garland's DINOMIT model of carcinogenesis, vitamin D deficiency plays a role in arresting carcinogenesis at various levels. This finding could pave the way for including vitamin D in the adjuvant treatment regime of epithelial cancers (2). Vitamin D and its metabolites, if linked with specific cancer types, can be used as nonspecific markers of aggressiveness. Adequate vitamin D levels are beneficial not only to decreasing the incidence of malignancy but also in a multitude of various health conditions, autoimmune conditions, infections, and chronic medical disorders. Food fortification with vitamin D could be considered if future large-scale studies show a similar picture.

In the current study, vitamin D insufficiency in the control group was around 35%, which was much lower than the range reported in Indian literature (5). Large-scale population-based studies have shown that serum 25-OH vitamin D levels are inversely associated with breast cancer risk in a concentration-dependent fashion (7). Garland and cols. reviewed around 3000 articles investigating the association of vitamin D and its metabolites with cancer (2). Imtiaz and cols., in their study on Pakistani women, found that 95.6% of women with breast cancer and 77% of healthy women were deficient in vitamin D, which was statistically significant (*p* < 0.001) (3). Harinarayan and cols. in their study found that 70% of South Indian women are deficient in vitamin D, 29% have insufficient levels, and only 1% has sufficient vitamin D levels (5).

Previous studies have shown a correlation between high BMI and low vitamin D levels (8). Since the BMI of the patients and controls were matched in the present study, the effect of BMI as a confounding variable has been suppressed to prevent a spurious association of vitamin D insufficiency with cases. In the current study, the postmenopausal women in the cases had low mean serum 25-OH vitamin D levels compared to the premenopausal group; however, the difference was not statistically significant. Using a statistical adjustment for menopausal status is advocated if the difference in the mean 25-OH vitamin D levels significantly differ in both the groups. Earlier studies have shown that postmenopausal women may have a relatively low 25-OH vitamin D concentration (9).

## Association of 25-OH vitamin D levels with breast cancer patients

The clinical stages of tumors did not bear any statistically significant relationship with the serum 25-OH vitamin D levels in this study. A similar finding was obtained in the study by Imtiaz and cols. on Pakistani women (3). The commonest presentation of breast cancer is a painless breast lump. Most of the people in rural India tend to neglect these symptoms. As a result, patients with advanced clinical stage at presentation are a common occurrence in the Indian setting. Because patients’ health-seeking behavior is a confounding factor in assessing the clinical staging at presentation, it may not be expected to show any association with vitamin D levels (10).

Patients with poorly differentiated breast cancer had lower vitamin D levels than patients with moderately and well-differentiated tumors. The above finding has been explained by previous studies in which it was demonstrated that vitamin D metabolites inhibit proliferation and promote apoptosis and differentiation (11).

Chollet and cols. showed that NPI, which was used to prognosticate primarily operable breast cancer in the adjuvant setting, retains its prognostic value in the neoadjuvant setting also (12). When the NPI prognostic groups were reorganized to form two groups - namely Group I (excellent and good prognosis) and Group II (moderate I, moderate II, and poor prognosis) - it was found that Group I had statistically significant higher serum 25-OH vitamin D levels than Group II (*p* = 0.008).

NPI is directly proportional to tumor grade. In the present study, the serum 25-OH vitamin D levels of the patients at diagnosis have a statistically significant association with tumor grade. Hence, the relationship of serum 25-OH vitamin D levels with various grades of tumors might contribute to its association with NPI. Because NPI has been used as a marker for prognosis, it could be assumed that a low serum 25-OH vitamin D level is one of the markers of poor prognosis.

Gene expression profiling in breast cancer has resulted in the identification of several major breast cancer subtypes. The best characterized of these subtypes are luminal A, luminal B, *HER2,* and basal-like carcinoma. Immuno-phenotyping with ER, PR, and *HER2* biomarkers can be used as a surrogate for molecular category of breast cancer (13–15). Luminal sub-group constitutes approximately 70% of invasive breast cancers. It is characterized by high expression of hormone receptors that are found more in luminal A. Luminal B tends to be at a higher histological grade than luminal A. Luminal B, sometimes called triple positive breast cancer, is more likely to have lymph node metastasis and may respond more to chemotherapy. Some luminal B tumors over-express *HER2.* These tumors are slow growing, respond well to hormone therapy and usually has a better prognosis (14,15,20). *HER2* constitutes around 15% of invasive breast cancers which are characterized by *HER2* over-expression, gene amplification and low expression of hormone receptors. These tumors have relatively poorer prognosis. Basal tumors are subgroup of the so-called triple-negative breast cancers. It is characterized by low expression of hormone receptors and *HER2* genes. Basal epithelial genes and basal cytokeratins (EGFR, CK 5/6) are highly expressed. The BRCA-associated breast cancers fall in this group. It has a poorer prognosis (15).

Using IHC findings as surrogate markers, luminal A and luminal B were grouped as luminal type breast cancer. *HER2 neu* and basal-like groups were classified as non-luminal breast cancer. Like other study results, patients with luminal types of breast cancer had higher serum 25-OH vitamin D levels than patients with the non-luminal types (14–16). The presence of lymphovascular invasion in the pathology specimen is a marker of poor prognosis. In the current study, the difference between serum 25-OH vitamin D levels among tumors with and without lymphovascular invasion was not statistically significant (*p* = 0.360). A similar observation was reported in the study by Imtiaz and cols. (3). Because the presence of tumor emboli within vascular spaces within the realm of a tumor is a common finding, rigorous sampling techniques are required to prevent this finding from being mistaken for the presence of true lymphovascular invasion (15).

Estrogen receptor negativity is one of the poor prognostic indicators in breast cancer (13). The antiproliferative action of vitamin D in breast cancer could partly be mediated by its role in down-regulating estrogen receptor abundance in breast cancer (7,11). Because estrogen causes increased growth of breast cancer, this anti-estrogenic effect of vitamin D is of much interest.

Vitamin D induces estrogen receptor expression in ER-negative breast cancers, thereby restoring their response to anti-estrogens, according to Santos-Martinez and cols. (16). The above-mentioned fact could form the way for a possible new therapeutic approach of adding vitamin D to potentiate adjuvant hormonal therapy in the future. Imtiaz and cols. could not demonstrate a correlation of vitamin D levels with parameters like histological grade of a tumor and estrogen receptor status (3). The present study, however, was able to document a statistically significant correlation between grade of a tumor (*p* = 0.001), estrogen receptor status (*p* = 0.045), luminal and non-luminal breast cancer (*p* = 0.045), and serum 25-OH vitamin D levels.

A unique feature of this study is that the study population was from the southern part of India, where studies comparing breast cancer and serum 25-OH vitamin D levels are scarce in this population. The study and control groups were matched meticulously in various aspects like age, BMI, menstrual status, parity, and co-morbidities like diabetes and hypertension. According to Chollet and cols., NPI also retains its prognostic value in the post-chemotherapy setting (12). In the present study, NPI had been used as an indicator of poor prognosis.

Robsahm and cols. analyzed the published literature on the inverse association of vitamin D on cancer survival and evaluated the possible reverse causation (17). It has been shown that the duration of the cancer, chemotherapy, and other modalities of cancer treatment may have an adverse effect on vitamin D levels. It is plausible that the inverse association of lower vitamin D levels and cancer could be due to the adverse effect of cancer treatment on vitamin D levels with a potential reverse causation. Because the duration and severity of cancer, various cancer treatments, and time of serum sample collection play a vital role in establishing the temporal association, the present study excluded patients who were diagnosed elsewhere or partially treated at outside hospitals. To avoid the possible adverse effect of chemotherapy and hormonal therapy on vitamin D levels, only newly diagnosed breast cancer patients were included in the present study.

Despite the different stages of cancer, one's population group and different sites of cancer may have an effect on vitamin D levels. Robsahm and cols. concluded that the association of lower vitamin D levels and inferior cancer survival is consistent even after adjusting for these confounders. Because a possibility of introducing vitamin D supplementation for the general population exists when extrapolating the study results, until strong evidence to prove the inverse association of vitamin D levels and breast cancer is available from the carefully structured prospective trials, the potential reverse causation should be considered when discussing this important subject. Results from the VITamin D and OmegA-3 TriaL (VITAL) on the efficacy of vitamin D supplementation in the prevention of cancer are much awaited and could possibly answer the above unsolved issue (18).

A limitation of this study was that patients who attended the hospital were selected based on convenient sampling. The current study was not a community-based one and patients were not followed up. Because it was a one-time estimate of vitamin D levels, it cannot be taken as an indicator of the duration of exposure to low serum levels of vitamin D that may have contributed to the increased risk for breast cancer. The sample size included in the present study is relatively small due to limited resources. A population-based study with a larger sample may provide better insight into this important subject.

Future studies may also include detailed assessment of nutritional status and quantification of sunlight exposure (time of exposure, duration) and skin color that would enable examination of the association of the above factors with serum 25-OH vitamin D levels. Large-scale studies that measured serum parathormone, serum calcium, bone mineral density, and serum 25-OH vitamin D levels in the Indian setting will help in defining a vitamin D classification in the Indian setting. The response to chemotherapy in patients in the neoadjuvant setting and its correlation, if any, with the vitamin D values could be studied. If a positive correlation were found, then it would pave the way for using vitamin D levels as a prognostic marker for assessing the response to chemotherapy (19).

In conclusion, higher grades of tumors had lower serum 25-OH vitamin D levels than lower grades. Luminal types of breast cancer had higher serum 25-OH vitamin D levels than the non-luminal types. Patients with estrogen receptor positivity had higher serum 25-OH vitamin D levels than those with absence of estrogen receptor. It was found that patients with excellent and good NPI prognoses had statistically significant higher serum 25-OH vitamin D levels than the moderate and poor NPI prognoses groups, whereas menopausal status, the clinical stage at presentation, and lymphovascular invasion did not show a statistically significant correlation with serum 25-OH vitamin D.
